# Prenatal Chemosensory Learning by the Predatory Mite *Neoseiulus californicus*

**DOI:** 10.1371/journal.pone.0053229

**Published:** 2012-12-26

**Authors:** Paulo C. Peralta Quesada, Peter Schausberger

**Affiliations:** Group of Arthropod Ecology and Behavior, Division of Plant Protection, Department of Crop Sciences, University of Natural Resources and Life Sciences, Vienna, Austria; Royal Holloway University of London, United Kingdom

## Abstract

**Background:**

Prenatal or embryonic learning, behavioral change following experience made prior to birth, may have significant consequences for postnatal foraging behavior in a wide variety of animals, including mammals, birds, fish, amphibians, and molluscs. However, prenatal learning has not been previously shown in arthropods such as insects, spiders and mites.

**Methodology/Principal Findings:**

We examined prenatal chemosensory learning in the plant-inhabiting predatory mite *Neoseiulus californicus*. We exposed these predators in the embryonic stage to two flavors (vanillin or anisaldehyde) or no flavor (neutral) by feeding their mothers on spider mite prey enriched with these flavors or not enriched with any flavor (neutral). After the predators reached the protonymphal stage, we assessed their prey choice through residence and feeding preferences in experiments, in which they were offered spider mites matching the maternal diet (neutral, vanillin or anisaldehyde spider mites) and non-matching spider mites. Predator protonymphs preferentially resided in the vicinity of spider mites matching the maternal diet irrespective of the type of maternal diet and choice situation. Across treatments, the protonymphs preferentially fed on spider mites matching the maternal diet. Prey and predator sizes did not differ among neutral, vanillin and anisaldehyde treatments, excluding the hypothesis that size-assortative predation influenced the outcome of the experiments.

**Conclusions/Significance:**

Our study reports the first example of prenatal learning in arthropods.

## Introduction

Prenatal or embryonic learning, defined as behavioral change following experiences before birth, i.e. *in ovo* or *in utero*, (e.g. [1]), is a widespread type of maternal effect. Most research on prenatal learning to date has been carried out with mammals, particularly humans, rats and dogs (e.g. [2]–[6]). One of the first terms employed to describe prenatal learning in humans was fetal conditioning [7], [8]. For example, Spelt [8], used vibrotactile equipment to condition human embryos *in utero* during the last two months of gestation. Prenatal learning has also been observed in birds [9], [10], amphibians [11]–[13] and molluscs [14] in various ecological contexts, but, strikingly, there have been no reports of prenatal learning in arthropods. Several papers on prenatal/embryonic learning (e.g. [2], [14]) refer to the review by Caubet et al. [15] as documentation of prenatal learning in insects. However, this study [15] reports pre-imaginal, i.e. larval (postnatal), rather than embryonic learning by insects.

Prenatal learning is not restricted to a specific sensory modality but may occur with auditory, visual, or chemosensory cues. Auditory prenatal learning is well-known from birds. For example, prenatal exposure to maternal vocalizations may serve to canalize the formation of species-specific auditory preferences in ducklings [9] and quail [16]. Visually exposing cuttlefish, *Sepia officinalis*, embryos to crabs before hatching resulted in a visual preference of cuttlefish juveniles for crab prey after hatching [14]. Chemosensory prenatal learning is the most widely documented type of prenatal learning. Numerous studies have addressed prenatal chemosensory learning in humans by exposing the embryos to different flavors, which affected their food preferences during childhood (e.g. [3], [17]). Prenatal flavor learning through the maternal diet is associated with subsequent postnatal preference for this flavor in many other mammals, such as pigs [18], [19], mice [17], rabbits [20], rats [4], [21], dogs [5], cats [22] and lambs [1]. For example, prenatal exposure of dog embryos to aniseed flavor through the maternal diet induced a feeding preference for this flavor in pups [5]. Similarly, the olfactory preference of neonatal kittens was shaped by prenatal exposure to cheese flavor through the maternal diet [22]. Prenatal chemosensory learning also occurs in birds (e.g. [10], [23]) and amphibians (e.g. [12], [13]). For example, Sneddon et al. [23] examined how prenatal exposure to strawberry flavor affects chemosensory preferences of newly hatched chickens. Naïve chickens avoided the strawberry flavor whereas chickens prenatally exposed to strawberry flavor preferred this flavor over alternative options. Mathis et al. [12] and Ferrari and Chivers [13] showed that amphibians are able to learn the chemical features of possible future predators during embryonic development. Ferrari and Chivers [13] exposed woodfrog, *Rana sylvatica*, embryos to different concentrations of injured conspecific tadpole cues paired with the odor of a predatory salamander, *Ambystoma tigrinum*. Tadpoles exposed to the predator odor paired with high concentrations of injured tadpole cues during embryonic development responded more strongly to the salamander, suggesting threat-sensitive learning.

Based on the striking lack of knowledge about prenatal learning and its relevance to foraging behavior in arthropods, here we investigate prenatal chemosensory learning in the plant-inhabiting predatory phytoseiid mite *Neoseiulus californicus* (McGregor). Many predatory mites, including *N. californicus*, are important natural enemies of herbivorous mites (such as spider mites) and insects (e.g. thrips), and are used in biological control in diverse agricultural ecosystems such as orchards, vineyards and greenhouse crops around the world [24]–[26]. Whilst *N. californicus* is a diet generalist it prefers tetranychid spider mites as food, but also consumes other mite species, small insects such as thrips, pollen and plant exudates (reviewed in [27]). All phytoseiid mites lack eyes and are therefore heavily reliant on chemosensory perception (e.g. [28], [29]). In foraging contexts, phytoseiid mites orient themselves mainly to chemicals released by their prey and its host plant (e.g. [28], [30]). Like all phytoseiid mite species, *N. californicus* has five life-stages (egg, larva, protonymph, deutonymph and adult) with the protonymph being the first feeding stage [31]. *N. californicus* has recently been shown to be able to learn in the very early postnatal stages, in the larval and early protonymphal stage, in foraging contexts [32].

Our general objective was to find out whether the predatory mite *N. californicus* is capable of prenatal chemosensory learning, experimentally assessed by embryonic exposure to two flavors, the phenolic aldehydes vanillin and anisaldehyde, incorporated into the eggs through a vanillin- or anisaldehyde-flavored maternal diet. Neither flavor has any ecological or evolutionary significance for the spider mites or their predators. Hence these flavors should be considered completely novel. Our specific objectives were to examine the residence and feeding preferences of *N. californicus* protonymphs, which had experienced the flavor profile of the maternal diet in the embryonic stage, in binary choice situations with spider mites matching the flavor of the maternal diet and spider mites with a different flavor. To exclude the explanation that possible behavioral alterations of the predator protonymphs were due to size effects induced by the different flavor treatments, such as size-assortative predation [33], we measured the body size of spider mites fed on differently flavored bean plants and the eggs produced by predatory mite females fed on differently flavored spider mites.

## Materials and Methods

### Origin and rearing of mites

*Neoseiulus californicus* used in experiments were derived from a laboratory-reared population founded with specimens originally obtained from Biotactics, California, USA via Koppert BV, The Netherlands [34]. The predator population was maintained on an artificial arena consisting of a plastic tile (15×15 cm) placed on a moist foam cube (15×15×6 cm) inside a rectangular plastic box (25×25×6 cm), half-filled with water. The edges of the tile were covered by moist tissue paper to confine the predators to the arena. The predators were fed two-spotted spider mites, *Tetranychus urticae*, by adding detached leaves of common bean (*Phaseolus vulgaris*) infested with *T. urticae* onto the arena every 3 to 4 days. *T. urticae* used to maintain the stock population of the predatory mites was reared on neutral bean plants (i.e. plants not enriched with any flavour).

### Pre-experimental procedure

The pre-experimental procedure was divided into three phases. The first phase comprised growth of the host plants in a system that allowed absorption of the vanillin and anisaldehyde flavors and their incorporation in the plant tissue. Vanillin (4-hydroxy-3-methoxybenzaldehyde: minimum purity 99%) and anisaldehyde (4-methoxybenzaldehyde: minimum purity 98%) were both obtained from Alfa Aesar, Karlsruhe, Germany. Plants were grown in a mixed substrate of sand and peat moss and watered every other day with 100 ml nutrient solution enriched with either vanillin or anisaldehyde or no flavor added (neutral). We used concentrations of 2.5 g vanillin or 0.5 ml anisaldehyde per liter of nutrient solution, which were determined in pilot experiments to not cause any adverse visible symptoms in the bean plants.

In the second phase, *T. urticae* to be used as prey for the predatory mites were reared on plants containing either vanillin or anisaldehyde or no added flavor (neutral) in their tissues. After growing the first trifoliate leaves, plants were infested with spider mites taken from the mass-rearing on neutral bean plants. The spider mites were fed on plants flavored with vanillin or anisaldehyde or on neutral plants between 4 and 30 days before being used as prey in experiments. Spider mites fed on plants flavored with vanillin or anisaldehyde or on neutral plants are hereafter termed vanillin, anisaldehyde and neutral spider mites.

In the third phase, gravid *N. californicus* females were fed on vanillin or anisaldehyde or neutral spider mites to lay eggs giving rise to protonymphs used in choice experiments. Spider mites reared on vanillin-flavored, anisaldehyde-flavored or neutral bean plants were collected separately in Petri dishes using a fine brush and then transferred to acrylic cages. Each cage consisted of a circular chamber (15 mm diameter) drilled into an acrylic glass plate (3 mm thick), closed at the bottom by a fine mesh and on the upper side by a glass slide secured with two metal clamps [35]. All cages were cleaned with 75% ethanol before use. Each cage was first loaded with 20 to 30 mixed spider mite life-stages (from eggs to adults) and then one gravid *N. californicus* female, randomly withdrawn from the stock population, was added. Inside the cages, the predatory mite females were fed with either neutral, vanillin or anisaldehyde spider mites for 3 days with prey replenished as needed. On the 3^rd^ day the predator females were transferred to a new empty cage for oviposition. On the 4^th^ day eggs laid within this cage were collected and each one was singly transferred to another empty cage. Females commonly produced only one egg on the 4^th^ day due to starvation. The inter-cage transfers were carried out to avoid any external contamination of the egg chorion with vanillin or anisaldehyde molecules.

The predator larvae hatched after ∼48 hours, and after another 24 hours the larvae molted to protonymphs, which were then used in the choice experiment. Both larvae and protonymphs were not fed and did not have any contact with prey before the choice experiments took place. The cages were stored in different boxes in environmental chambers at 25±1°C, 60±5% RH and a photoperiod of 16∶8 h L∶D.

### Choice experiment

Each *N. californicus* protonymph was given a choice between spider mites fed on the same type of plant (neutral, or vanillin- or anisaldehyde-flavored) as the spider mites fed on by their mothers (hereafter termed matching spider mites) and spider mites fed on one of the other two types of plant (hereafter termed non-matching spider mites). In total, six different combinations of protonymphal origin (produced by mothers fed on neutral, vanillin or anisaldehyde spider mites; hereafter termed neutral, vanillin and anisaldehyde protonymphs) and choice alternative (two for each protonymphal origin) with 24 replicates per combination (i.e. 6×24 protonymphs) were assayed. The choice experiments were carried out using T-shaped acrylic cages, laser-cut into 3 mm thick acrylic plates, composed of three circular chambers interconnected by a 2 mm wide aisle [36]. The two larger chambers (15 mm ø), located at either end of the horizontal bar of the T, contained the spider mites, while the smaller cavity (5 mm ø) at the bottom end of the vertical bar of the T was used as release point of the *N. californicus* protonymph. Before releasing the protonymph in the cage, each of the two larger chambers was filled with a similar mass (∼70 µg) of freshly killed neutral, vanillin, or anisaldehyde spider mites (predominantly eggs, larvae, and nymphs). The spider mites were killed by freezing for ∼45 min at −20°C immediately before the experiment. The spider mites were killed to avoid mixing of the two types presented during each choice experiment, and to avoid any inadvertent influence of their behavior, possibly changed by the differently flavored host plants, on their attractiveness to the predators. After releasing the predator protonymphs, the cages were closed on the upper side with a glass slide secured with two metal clamps. The cages were checked for the location (either inside one of the large chambers containing spider mites or elsewhere in the cage (considered the neutral zone)) and first feeding of the protonymph every 20 minutes for 140 minutes in total. Each predator protonymph and each cage was used only once in experiments.

### Size of prey and predators

To determine the size of the spider mites fed on differently flavored bean plants (neutral, vanillin, anisaldehyde) and the predators fed with differently flavored spider mites (neutral, vanillin- or anisaldehyde-flavored), respectively, the length (from the posterior end of the idiosoma to the tip of the gnathosoma) and width of spider mite larvae, nymphs and adult females, and the length and width of the predator eggs were measured. Predatory mite egg size positively correlates with their body size [37]. Twenty replicates were measured for each life stage and treatment. To take these measurements, the spider mites and predator eggs were embedded in a drop of Hoyer's medium [38] covered by a cover slip on a microscope slide. All measurements were carried out under a transmitted light microscope with an eyepiece graticule. After measuring the mites, the scale of the eyepiece graticule was calibrated using a stage micrometer and the measurements converted into µm. Length and width of the predator eggs were used to calculate egg volume, which resembles in shape a spheroid, according to the equation:
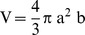
where *V* is the spheroid volume, *a* is the width and *b* is the length of the egg.

### Statistical analyses

SPSS 15.0.1 (SPSS Inc., Chicago, IL, USA) was used for all statistical analyses. Separate Wilcoxon signed rank tests were used to analyze the residence preference (with matching or non-matching spider mites) of the protonymphs within each of the six combinations of protonymphal origin (neutral, vanillin or anisaldehyde) and type of non-matching spider mites. A generalized linear model (GLM; count of events, binomial distribution, identity link) was used to analyze the influence of protonymphal origin and type of non-matching spider mites nested within protonymphal origin on residence preference of the protonymphs (with matching or non-matching spider mites). For the Wilcoxon signed rank tests and GLM, the data gathered over time were aggregated for each protonymph, i.e. the number of times each protonymph was found in the chamber containing matching spider mites, out of all observations of residence in the chambers with matching or non-matching spider mites, was used for analyses. Residences in the neutral zone were excluded from analyses. Binary logistic regression using protonymphal origin as covariate was used to test the feeding preference (first fed on matching or non-matching spider mites) of the protonymphs. Separate analyses of variance (ANOVAs) were carried out to compare the body length and width within each spider mite life-stage and the egg volume of the predatory mites among treatments (neutral, vanillin, anisaldehyde).

## Results

### Choice experiment

**Figure 1:**
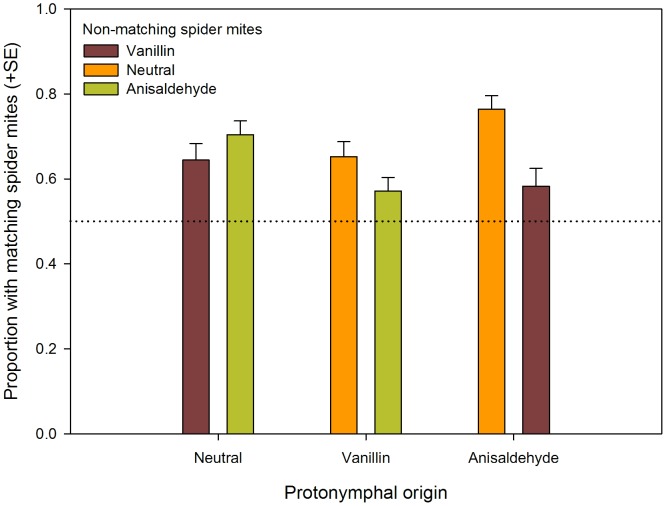
The effects of prenatal learning on residence preference of predatory mite protonymphs. Mean (± SE) proportion of *N. californicus* protonymphs preferentially residing in the vicinity of spider mites matching the type in their maternal diet. Protonymphs originated from mothers fed on neutral, vanillin- or anisaldehyde-flavored spider mites and were given a binary choice between dead spider mites matching the maternal diet and non-matching spider mites (*n* = 24 for each of the six choice situations).

Wilcoxon signed rank tests revealed that predator protonymphs chose the chamber containing the spider mites matching the maternal diet (neutral, vanillin- or anisaldehyde-flavored) significantly more often, irrespective of their origin (neutral, vanillin, anisaldehyde) and the type of non-matching spider mites offered (*Z*<−2.037, *n* = 24, and *p*<0.05 for each of the six choice situations; figure 1). GLM revealed that preferential residence in the chamber containing matching spider mites did not differ among protonymphal origins (*Wald χ_2_^2^* = 3.946, *p* = 0.139). The significant effect of type of non-matching spider mites (neutral, vanillin, anisaldehyde) nested within protonymphal origin (*Wald χ_3_^2^* = 13.184, *p* = 0.004) indicated variation in strength of preference within protonymphal origins (neural, vanillin, anisaldehyde). Scrutiny of the parameter estimates showed that this effect was only due to a significant difference between the two types of non-matching spider mites in anisaldehyde protonymphs (*Wald χ_1_^2^* = 9.448, *p* = 0.002), while the type of non-matching spider mites did not influence the residence preference of neutral (*Wald χ_1_^2^* = 1.347, *p* = 0.246) and vanillin (*Wald χ_1_^2^* = 2.389, *p* = 0.122) protonymphs. The preference of anisaldehyde protonymphs for anisaldehyde spider mites was more pronounced when given a choice between anisaldehyde and neutral spider mites than when given a choice between anisaldehyde and vanillin spider mites (figure 1).

In total, 25 protonymphs were observed feeding (1 neutral, 10 anisaldehyde, 14 vanillin). The neutral protonymph was observed feeding on neutral spider mites but was excluded from analysis because it was the only one for this protonymphal origin. In 17 of the remaining 24 cases the protonymphs first fed on spider mites matching the maternal diet, which was significantly higher than random expectation (binary logistic regression; *Wald χ_1_^2^* = 3.904, *p* = 0.048). Type of maternal diet (vanillin or anisaldehyde) did not influence the feeding preference of the protonymphs for the prenatally experienced spider mite flavor (*Wald χ_1_^2^* = 0.681, *p* = 0.409).

### Size of prey and predators

Maternal diet had no influence on the volume of the predatory mite eggs giving rise to protonymphs used in experiments (mean ± SE; neutral 0.0180±0.0004, vanillin 0.0179±0.0003, anisaldehyde 0.0178±0.0003 µm; ANOVA: *F_2,57_* = 0.111, *p* = 0.895). Similarly, the body size of spider mite larvae (ANOVA: length *F_2,57_* = 0.202, *p* = 0.818; width *F_2,57_* = 1.357, *p* = 0.266), nymphs (length *F_2,57_* = 0.105, *p* = 0.901; width *F_2,57_* = 0.061, *p* = 0.941) and adult females (length *F_2,57_* = 0.482, *p* = 0.620; width *F_2,57_* = 1.341, *p* = 0.270) did not differ among host plants (neutral, vanillin- or anisaldehyde-flavored: table 1).

**Table 1:** Body length and width (µm; mean ± SE) of spider mites fed on bean plants (*P. vulgaris*) flavored with vanillin or anisaldehyde or non-flavored (neutral).

Life stage	Treatment	Length (µm)	Width (µm)
Larva	Anisaldehyde	0.1753±0.0030	0.1376±0.0008
	Neutral	0.1723±0.0035	0.1367±0.0012
	Vanillin	0.1745±0.0039	0.1389±0.0009
Nymph	Anisaldehyde	0.3154±0.0156	0.2040±0.0064
	Neutral	0.3220±0.0168	0.2035±0.0070
	Vanillin	0.3112±0.0182	0.2065±0.0066
Adult female	Anisaldehyde	0.5635±0.0150	0.3010±0.0088
	Neutral	0.5454±0.0137	0.2827±0.0068
	Vanillin	0.5613±0.0141	0.2965±0.0088

Treatment did not affect the length and width in any of the life stages: larva, nymph or adult female (ANOVAs: *p*>0.05 for each measurement).

## Discussion

Our study provides evidence that the foraging preferences of juvenile predatory mites, *N. californicus*, can be significantly influenced by prenatal chemosensory experiences, through exposure of the embryos to chemicals present in the eggs due to maternal diet. To the best of our knowledge our study is the first to show prenatal learning in arthropods, adding *N. californicus* to the growing list of animal species in which prenatal chemosensory learning has been observed. The occurrence of prenatal chemosensory learning across diverse animal taxa suggests widespread adaptive significance of this ability. The ability of embryos to perceive and correctly interpret chemosensory cues associated with the maternal diet could, for example, confer adaptive advantages in exploiting this diet after hatching through reduced searching times and quicker diet recognition and acceptance.

Proximately, we assume that the predatory mite females incorporated the chemical signatures of their prey, including the anisaldehyde and vanillin flavors, into their eggs. Eventually, during choice experiments, the protonymphs displayed a preference for the spider mites with the chemical signature they had experienced as embryos. In vertebrates this chemical information exchange has been explained by the transfer of nutrients, metabolites and flavors in the maternal diet to the embryo inside the womb [2], [19], [39] or egg [11], [12], [23]. Although embryonic development of mites [40], and arthropods in general [41], occurs under completely different conditions than that of vertebrates, our study suggests that analogous chemical transfers from the mother to the embryo take place in *N. californicus*. We suggest that *N. californicus* females transferred the chemical signatures, including the flavor molecules, of the prey on which they fed during egg formation to the yolk, in addition to other nutrients essential for embryonic nourishment and development. The embryos perceived and learned these chemical signatures when developing inside the egg. Memory retention during development and molting [32], [42]–[44] through the maintenance of neurons formed in the embryonic stage allowed postnatal recognition of these molecules. During formation, the predator eggs were enclosed in the uterus nourished via cords [45] and, after laying, matured in prey-free environments. Thus, for our experiments we consider the possibility of postnatal learning via contamination of the egg chorion with the chemical signatures of the maternal prey, unlikely. Such contamination can be an issue with herbivores and parasitoids [46], which have a more intimate physical association with their hosts than true predators such as *N. californicus* have with their prey.

Preferential residence of the predatory mites close to the spider mites matching the maternal diet was evident in all choice combinations. The more pronounced preference by anisaldehyde predators for anisaldehyde spider mites when neutral spider mites were the alternative than when vanillin spider mites were the alternative, may be explained by the structural similarity of both phenolic aldehydes. Both chemicals are composed of a cyclic carboxylic acid (phenol) attached to the C1 aldehyde functional group, but differ slightly as anisaldehyde has a methoxy group in C4 and vanillin has an ether in C3 [47], [48]. Likewise, the preference of vanillin protonymphs for vanillin spider mites was also more pronounced, albeit not significantly, when offered neutral than when offered anisaldehyde spider mites as an alternative.

Across choice combinations, the protonymphs responded very specifically but qualitatively similar - always showing a preference for the spider mites matching the maternal diet (neutral, vanillin or anisaldehyde) - suggesting a specific associative learning mechanism rather than a general mechanism such as sensitization [49]. The use of dead spider mites excluded any effect due to behavioral alterations possibly induced by the differently flavored host plants. Size-assortative predation, i.e. the commonly observed positive correlation between predator and prey body sizes (e.g. [33]), can be excluded as a potential alternative explanation for the observed residence and feeding preferences. Neither treatment (neutral, vanillin or anisaldehyde) induced any size variation in prey and predators, respectively. Vanillin and anisaldehyde increased the propensity of the predator protonymphs to feed on spider mites during the experiment, indicating that these substances acted as feeding attractants or stimulants. Anisaldehyde acting as feeding attractant has also been observed in other herbivorous arthropods such as the western flower thrips, *Frankliniella occidentalis* [50]. Likewise, the feeding propensity of lambs was positively influenced by prenatal exposure to oregano flavor [1]. These observations support the idea of prenatal associative learning. Individuals prenatally exposed to the novel flavor associated this flavor with food, and recognized this learned cue later (after birth), while individuals lacking prenatal experience with the novel flavor did not have any additional cue to form such associations.

Our study provides a key example of prenatal learning in arthropods. However, future research should assess prenatal learning in other species of insects, spiders and mites to see how widespread this phenomenon is in arthropods,. Pursuing the questions whether and how prenatal learning effects induced by the maternal diet could be reinforced or changed by postnatal learning would be other promising routes of research. It would be of great significance to evaluate for how long memory of prenatal experiences can be maintained through ontogeny. In dogs the combination of both pre- and postnatal experiences may synergistically enhance the preference for a given food type [5]. Thus, it would be interesting to test whether pre- and postnatal exposures to the chemical signature of a given food type enhance the acceptance and/or preference of the predatory mites for this food type more than expected from either pre- or postnatal exposure alone [1], [51]. The finding that *N. californicus*, and possibly other generalist predators, are able to pre- and postnatally [32] learn the chemical signatures of their prey, and adjust their foraging behaviors accordingly, could be exploited in biological control. It should be possible to generate, through early conditioning, *N. californicus* lines that preferentially feed on, or more readily accept, a given prey type, which should improve the predators' performance in biological control of this prey. Conditioning might yield more selective predators and result in greater immediate suppression of key pest species.
